# Management of Severe Epithelial Ingrowth Following KLEx Using Crocodile-Forceps Extraction: Surgical Technique and Literature Review

**DOI:** 10.3390/jcm15176928

**Published:** 2026-09-07

**Authors:** Francesco D’Oria, Ilaria Lolli, Pasquale Puzo, Giovanni Petruzzella, Giovanni Alessio

**Affiliations:** Section of Ophthalmology, Department of Translational Biomedicine and Neuroscience, University of Bari, 70125 Bari, Italy

**Keywords:** epithelial ingrowth, keratorefractive lenticule extraction, corneal refractive surgery, anterior segment OCT

## Abstract

**Background**: Epithelial ingrowth is an uncommon but potentially vision-threatening complication after keratorefractive lenticule extraction (KLEx). Although this complication is well described after flap-based refractive surgery, its occurrence after flapless lenticule extraction remains rare and is mainly reported in isolated cases. This narrative review summarizes the available literature on epithelial ingrowth after KLEx, with particular attention to proposed mechanisms, clinical presentation, imaging findings, and surgical management. An illustrative case of severe epithelial ingrowth managed with crocodile-forceps extraction is also presented. **Methods**: A literature search was performed to identify published reports describing epithelial ingrowth after SMILE/KLEx. Relevant articles were reviewed and compared with regard to onset, suspected mechanism, clinical severity, treatment strategy, anatomical outcome, visual recovery, and recurrence. **Results**: The affected eye developed extensive epithelial ingrowth associated with a postoperative tunnel laceration and marked visual deterioration during the first postoperative week. Surgical clearance through the original incision was performed using crocodile forceps followed by copious interface irrigation. Approximately one hour after treatment, AS-OCT demonstrated complete clearance of the hyperreflective interface material, corneal topography showed marked regularization, and wavefront aberrometry demonstrated a reduction in overall OPD RMS from 1.21 to 0.58 µm and higher-order RMS from 1.03 to 0.37 µm at a 4 mm pupil. Visual acuity subsequently improved over the following postoperative days, with no recurrence during 6-month follow-up. **Conclusions**: Severe epithelial ingrowth after KLEx may require prompt surgical intervention when rapid progression and visual deterioration occur. In this case, crocodile-forceps-assisted interface clearance through the original incision was feasible and was associated with rapid anatomical and optical recovery without cap-to-flap conversion or chemical adjuvants. Further cases are required to determine the reproducibility, safety, and long-term outcomes of this approach.

## 1. Introduction

Epithelial ingrowth is a well-recognized complication after lamellar corneal refractive procedures, particularly laser-assisted in situ keratomileusis (LASIK), where epithelial cells can migrate into the flap interface and impair optical quality [1,2]. In contrast, keratorefractive lenticule extraction (KLEx) is a flapless procedure in which the lenticule is extracted through a small corneal tunnel, making the occurrence of epithelial ingrowth relatively rare. Reported cases are usually mild, localized, and asymptomatic, but when epithelial proliferation becomes extensive, it may compromise corneal transparency, induce irregular astigmatism, and cause significant visual loss [3,4].

The case described here is unique because of the massive, vision-threatening epithelial ingrowth occurring within a few days of surgery, in association with an early tunnel laceration after a small incision lenticule extraction (SMILE), managed with a novel surgical technique [5,6]. To our knowledge, only a limited number of cases of extensive ingrowth after SMILE have been reported, and few have provided detailed documentation of both the clinical course and surgical management [7]. The aim of this narrative review is to summarize the available evidence on epithelial ingrowth following KLEx, focusing on its proposed mechanisms, clinical presentation, diagnostic features, and management strategies. In addition, we present an illustrative case of severe, rapidly progressive epithelial ingrowth following SMILE, successfully managed with crocodile-forceps extraction. By integrating the clinical case with the published literature, this review seeks to clarify the therapeutic options available for this rare but potentially vision-threatening complication.

## 2. Case Report

**Figure 1:**
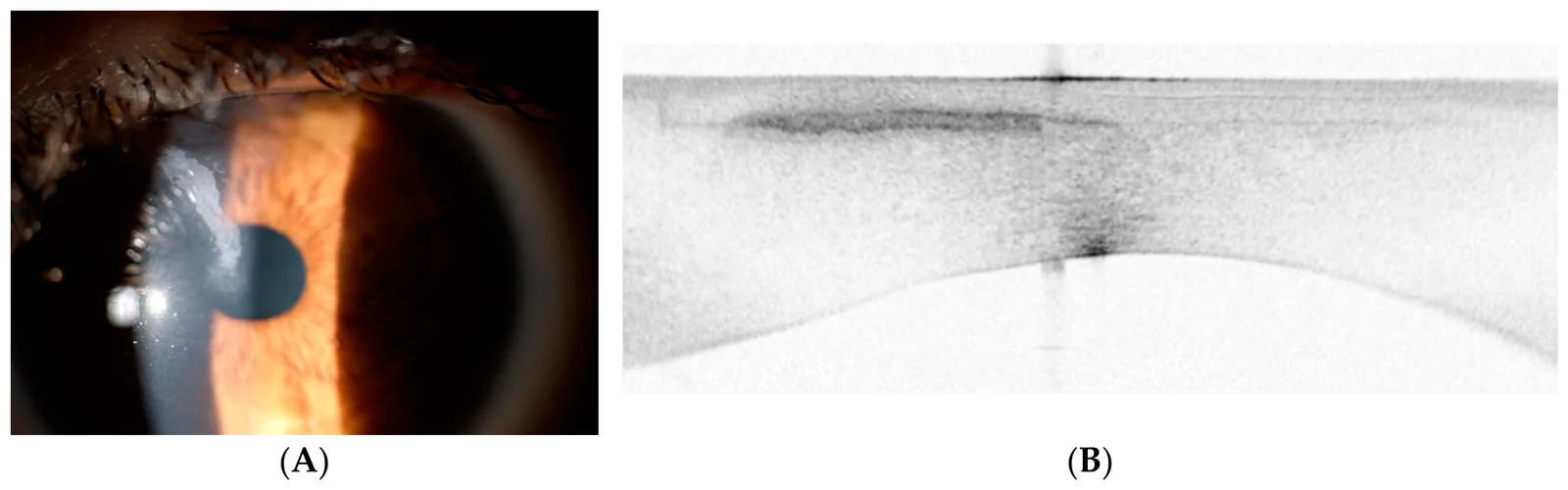
Pre-debridement slit-lamp biomicroscopy and anterior segment optical coherence tomography (AS-OCT) of the left eye. (**A**) Slit-lamp image showing a dense whitish opacity within the stromal interface, consistent with extensive epithelial ingrowth. (**B**) AS-OCT demonstrating a hyperreflective layer extending across the lenticule interface.

A 25-year-old female patient underwent bilateral keratorefractive lenticule extraction (KLEx) for the correction of myopia. Preoperative refraction was −3.00 D sphere with −1.00 D cylinder in the right eye and −2.50 D sphere in the left eye. Bilateral KLEX was performed using the VisuMax 800 platform (Carl Zeiss Meditec, Jena, Germany), with a 120 µm cap thickness, a 3.0 mm incision, and a 6.4 mm optical zone. The procedure was uneventful in the right eye. In the left eye, a minor tunnel laceration was recognized intraoperatively during the procedure, without an apparent epithelial defect or immediate impact on visual acuity. The right eye showed an excellent postoperative course, with uncorrected visual acuity (UCVA) of −0.1 LogMAR at one week, a regular topographic pattern, and clear anterior segment optical coherence tomography (AS-OCT) images. By the end of the first postoperative week, however, the patient reported a marked visual decline, with UCVA reduced to 0.5 LogMAR. Slit-lamp biomicroscopy revealed a dense, whitish opacity within the stromal interface, highly suggestive of epithelial ingrowth. Corneal topography demonstrated irregular astigmatism, and AS-OCT confirmed the presence of hyperreflective material spreading across the lenticule interface (Figure 1).

Given the rapid progression of the interface lesion and the associated visual deterioration, surgical intervention was performed under topical anesthesia. The original SMILE incision was used to access the lenticule interface, avoiding cap-to-flap conversion. The interface was carefully dissected through the existing incision until the epithelial proliferation was exposed. Crocodile forceps were then introduced through the incision and used to grasp the epithelial nests under high magnification. The tissue was removed progressively with gentle traction, with particular care taken to minimize fragmentation and avoid unnecessary manipulation of the stromal interface. After removal of the visible epithelial tissue, copious irrigation with balanced salt solution was performed to remove residual cellular debris and facilitate smooth stromal apposition. No cap-to-flap conversion and no chemical adjuvant, including mitomycin C, alcohol, or hydrogel sealant, were used. A bandage contact lens was placed. Postoperatively, the patient received topical netilmycin and desamethasone four times daily for one week (Video S1). The differential diagnosis of an early postoperative interface opacity included epithelial ingrowth, infectious keratitis, and sterile inflammatory interface reaction. Infectious or inflammatory complications were considered less likely because there were no clinical signs of active infection or significant intraocular inflammation, and the interface lesion showed the characteristic hyperreflective appearance and distribution observed on AS-OCT. The rapid and complete disappearance of the interface material following direct mechanical removal further supported the diagnosis of epithelial ingrowth. No microbiological sampling was performed because the clinical presentation did not suggest an infectious process.

**Figure 2:**
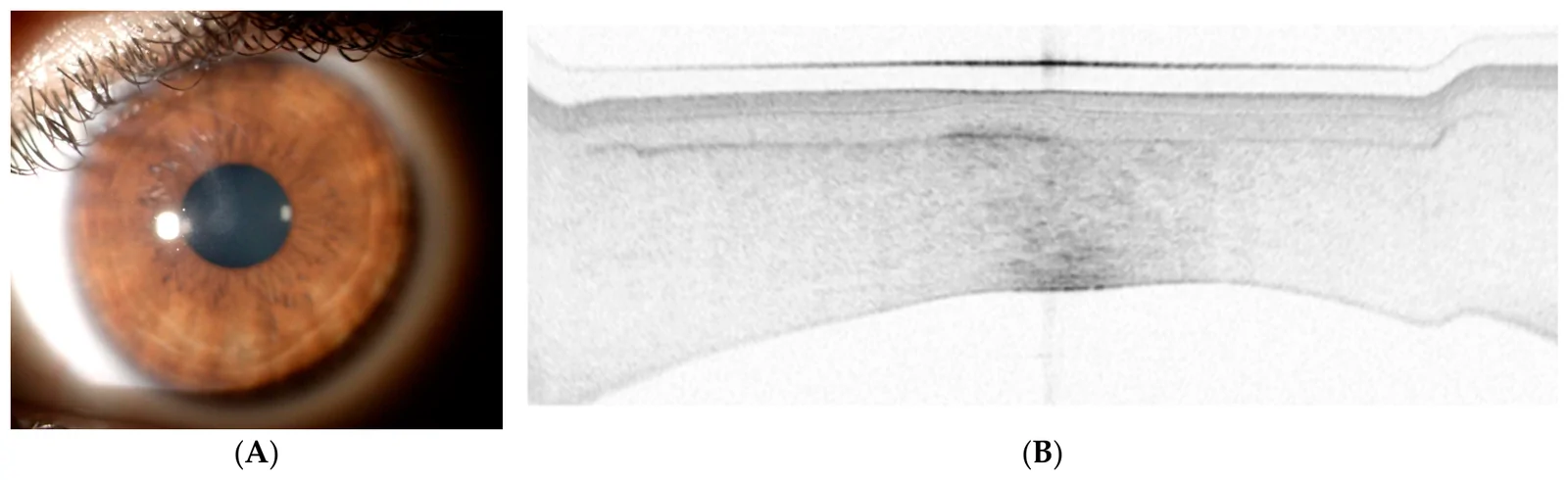
Post-operative slit-lamp biomicroscopy and anterior segment optical coherence tomography (AS-OCT) of the left eye following surgical debridement. (**A**) Slit-lamp image demonstrating complete resolution of the interface opacity, with restoration of corneal transparency. (**B**) AS-OCT confirming a clear, smooth lenticule interface with no residual hyperreflective material, consistent with full anatomical recovery.

The postoperative course was favorable. Immediate postoperative slit-lamp examination and AS-OCT demonstrated complete clearance of the previously visible interface opacity and restoration of interface transparency (Figure 2). Corneal topography and wavefront aberrometry were subsequently obtained approximately one hour after surgical clearance, providing an objective assessment of the immediate anatomical and optical changes.

Visual acuity improved progressively after surgical clearance. Although the anatomical and optical changes were already evident on the immediate postoperative imaging assessment, functional visual recovery continued over the following postoperative days. UCVA improved from 0.5 to 0.0 LogMAR. No recurrence of epithelial ingrowth was observed during the 6-month follow-up period.

**Figure 3:**
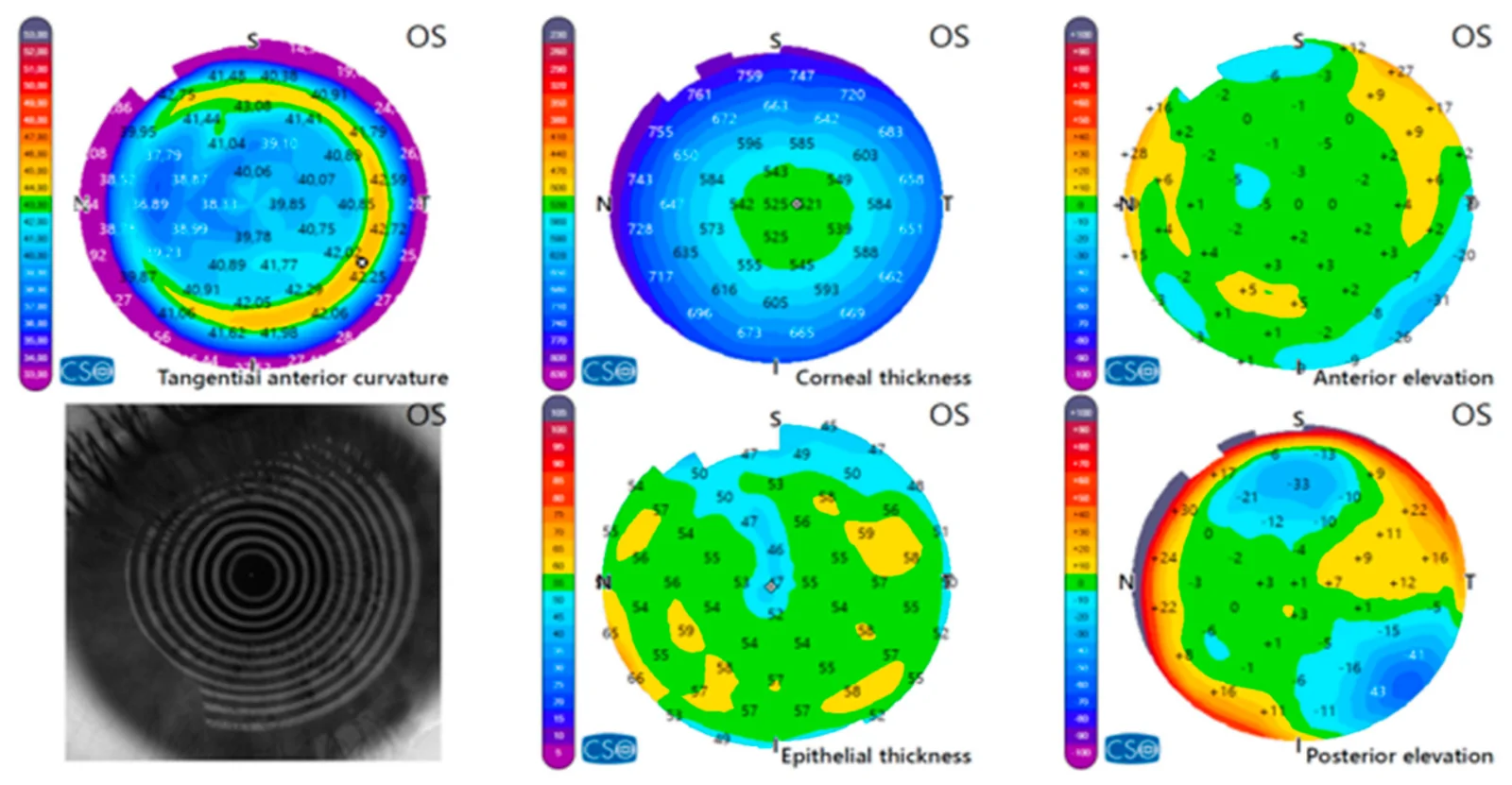
Corneal topography of the left eye immediately after surgical debridement, demonstrating restoration of corneal regularity after interface clearance.

After surgical debridement, topographic maps demonstrated a marked improvement in corneal regularity, with restoration of a smooth curvature profile and reduction in the topographic index values toward preoperative levels. Epithelial thickness mapping performed immediately after surgical debridement demonstrated focal epithelial thinning corresponding to the area previously occupied by the epithelial ingrowth. As the epithelial mapping was obtained immediately after interface clearance, before compensatory epithelial remodeling could occur, this finding is reported descriptively, and no inference regarding the underlying stromal contour was made based on epithelial thickness mapping alone (Figure 3).

**Figure 4:**
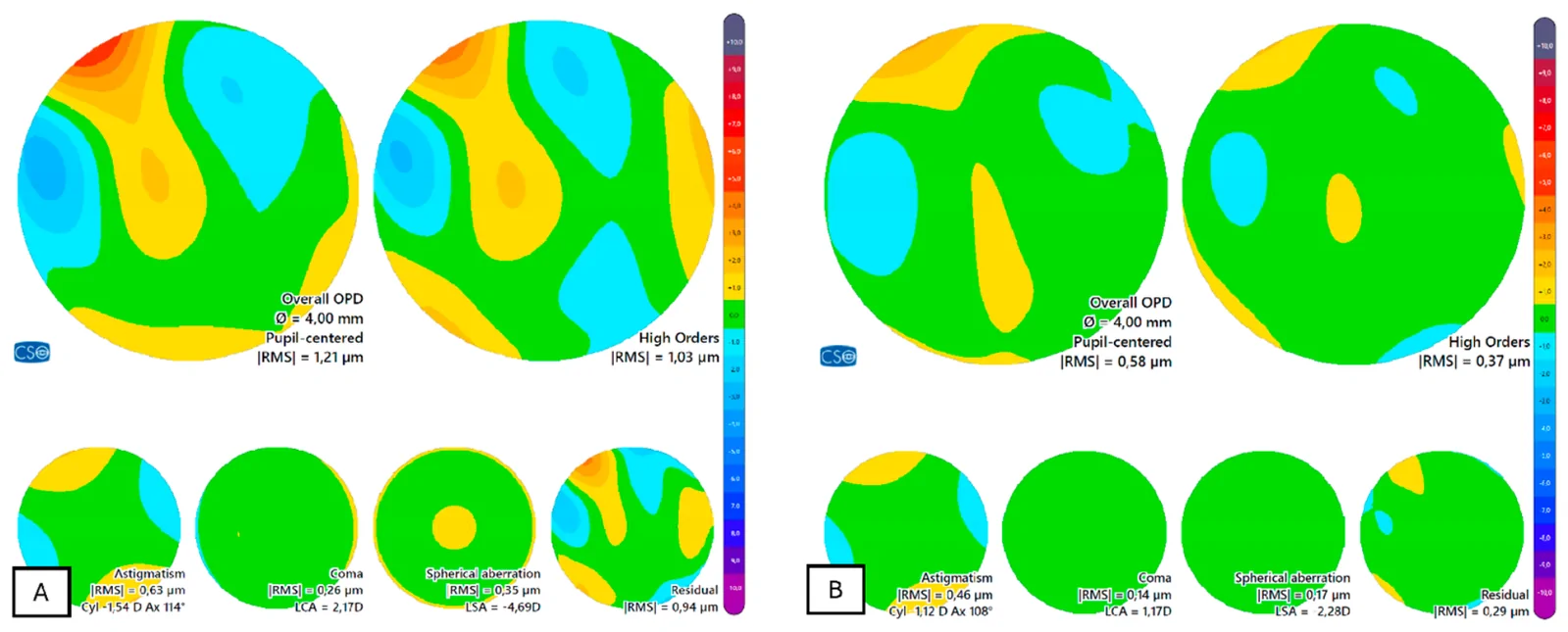
(**A**) Pre- and (**B**) post-operatory wavefront aberrometry comparison showing reduction in higher-order aberrations (coma, trefoil) following surgical treatment, consistent with visual recovery.

Wavefront aberrometry demonstrated marked optical improvement following surgical clearance. At a 4 mm pupil diameter, overall OPD RMS decreased from 1.21 µm before treatment to 0.58 µm approximately one hour after treatment, while higher-order aberration RMS decreased from 1.03 µm to 0.37 µm. Coma RMS decreased from 0.26 µm to 0.14 µm, astigmatism RMS from 0.63 µm to 0.46 µm, spherical aberration RMS from 0.35 µm to 0.17 µm, and residual RMS from 0.94 µm to 0.29 µm. These findings represent descriptive pre- and post-treatment changes in a single eye and are therefore not interpreted as statistically significant differences. The marked reduction in higher-order aberrations was consistent with the subsequent clinical improvement in visual function (Figure 4).

The right eye remained stable with excellent vision, underscoring the unilateral nature of the complication despite bilateral surgery.

## 3. Discussion

Epithelial ingrowth is a well-known complication of flap-based refractive procedures, particularly LASIK, where epithelial cells may migrate beneath the flap and proliferate within the interface [1]. In contrast, KLEx is considered less prone to this complication because it avoids the creation of a corneal flap. Nevertheless, epithelial cells can still access the interface through the small side-cut incision, especially when there is tunnel trauma, an intraoperative epithelial defect, or excessive manipulation during lenticule extraction [3,4,8].

The present case illustrates a rare but severe form of epithelial ingrowth after SMILE. The precise mechanism underlying epithelial ingrowth in this case remains uncertain. However, the minor tunnel laceration observed intra-operatively may have acted as a permissive pathway for epithelial cell migration into the stromal interface. This interpretation is consistent with previously reported incision-related cases, although direct intraoperative epithelial implantation, epithelial defects, and other mechanisms cannot be excluded. Given that the laceration was documented before the development of clinically evident extensive epithelial ingrowth, it represents the most plausible anatomical risk factor identified in the present case, but causality cannot be established [5,6,7]. The patient developed a rapid and massive proliferation that led to significant visual impairment within the first week. This clinical trajectory—characterized by early onset, aggressive growth, and central interface involvement—is considerably more severe than the asymptomatic or slowly progressive lesions described in most published reports [9], and aligns with cases in which incisional compromise appears to have acted as the primary permissive factor for epithelial cell migration.

A narrative literature search was performed to identify published reports describing epithelial ingrowth following keratorefractive lenticule extraction, including small incision lenticule extraction (SMILE) and related lenticule extraction techniques. The search was performed in PubMed/MEDLINE in June 2026. The search strategy included combinations of the terms “SMILE,” “small incision lenticule extraction,” “keratorefractive lenticule extraction,” “epithelial ingrowth,” “epithelial implantation,” “epithelial inoculation,” and related terms. Eligible publications included case reports, case series, and clinical reports describing epithelial ingrowth following KLEx/SMILE, with particular attention to clinical presentation, suspected mechanisms, imaging findings, treatment, recurrence, and visual outcomes. Publications not describing epithelial ingrowth after KLEx/SMILE were excluded (Table 1) [3,4].

Reported cases span a wide clinical spectrum, ranging from asymptomatic, non-progressive lesions discovered incidentally at routine follow-up to rapidly progressive ingrowth causing significant visual impairment and requiring urgent surgical intervention [5,6,7,10]. Identified risk factors include compromise of the side-cut incision (tunnel laceration or incisional tear), intraoperative epithelial defects, and excessive manipulation during lenticule delivery [3,4,8]. Additional pathogenetic mechanisms have been proposed: vertical epithelial gas breakthrough, whereby femtosecond laser-generated gas creates a transient fistula between the interface and the epithelial surface, has been implicated in at least one reported case [8], and more recently, the formation of opaque bubble layer (OBL) during lenticule dissection has been hypothesized to generate microchannels through Bowman’s layer, providing a further route for epithelial cell ingress [13]. In the present case, the minor tunnel laceration noted on postoperative day 1 most likely constituted the primary entry point, consistent with the incision-related mechanism described in the literature.

Management of interface epithelial ingrowth is challenging: several approaches have been described, each tailored to the extent and progression of the lesion.

When the ingrowth is small, stable, and does not involve the pupillary axis, observation alone may be appropriate, with intervention reserved for cases showing signs of progression [14].

In more symptomatic or progressive presentations, mechanical debridement has been employed, encompassing interface irrigation with balanced salt solution (BSS), blunt-spatula scraping, and direct removal of epithelial tissue using forceps—all performed without cap lifting when the lesion is accessible through the original incision [7,10].

For larger or more established ingrowths, cap-to-flap conversion via dedicated software (CIRCLE, Zeiss VisuMax) allows direct visualization and thorough debridement of both the stromal bed and the undersurface of the flap, with adjuvant mitomycin C (0.02%) employed in selected cases to reduce the risk of recurrence [5,6,12].

At the more severe end of the spectrum, cases refractory to repeated debridement have been managed with additional measures including hydrogel ocular sealant application to seal the incision and prevent epithelial re-entry [5]. The worst outcome in the available literature involved a patient requiring three sequential surgical procedures before resolution was achieved, with uncontrolled systemic disease identified as a potential contributing risk factor. Whichever approach is chosen, these options may carry risks of stromal toxicity, delayed healing, or interface haze [10,11,14].

In this case, surgical debridement was successfully achieved using crocodile forceps. This instrument allowed for precise grasping and removal of the friable epithelial nests without fragmentation, reducing the risk of residual cells remaining in the interface. The addition of copious irrigation further helped eliminate debris and restore smooth stromal apposition. Notably, no chemical adjuvants were required, and complete anatomical and visual recovery was achieved without recurrence.

The clinical features of the present case are consistent with the most severe presentations described in the literature, sharing the hallmark of incision-related compromise as the likely initiating event. Similarly, the decision to proceed with mechanical debridement without cap lifting aligns with approaches reported in comparable cases where the incision provided sufficient access to the interface. On the contrary, unlike most published cases, in which conservative irrigation alone, or blunt scraping with standard vitreoretinal microforceps, was used, the present report describes the use of crocodile forceps, whose jaw geometry allowed for en-bloc grasping of the friable epithelial tissue without fragmentation, minimizing the risk of residual cellular contamination. No chemical adjuvants were employed, in contrast to cases in the literature where mitomycin C, alcohol, or hydrogel sealant were added to prevent recurrence, thereby avoiding the associated risks of stromal toxicity and delayed healing [10,14].

What further distinguishes the present case is the early multimodal documentation of anatomical and optical recovery following surgical clearance. In the published cases identified in our literature search, postoperative outcomes were generally reported at intervals ranging from days to months, while immediate postoperative assessment with combined AS-OCT, corneal topography, and wavefront aberrometry was not described. In the present case, all three modalities demonstrated marked improvement within approximately one hour of interface clearance. Based on the publications identified through our literature search, this appears to represent the earliest documented multimodal imaging assessment of recovery following surgical treatment of epithelial ingrowth after KLEx. This observation should be interpreted descriptively rather than as evidence of superiority of the present technique.

In conclusion, crocodile-forceps-assisted interface clearance, combined with copious irrigation and performed without cap-to-flap conversion or chemical adjuvants, was feasible in this case of severe epithelial ingrowth following KLEx and was associated with rapid anatomical and optical recovery. The technique allowed direct removal of the epithelial proliferation through the original incision while avoiding a more extensive surgical approach. However, a single case cannot establish the safety, efficacy, superiority, or long-term recurrence profile of the technique. Crocodile-forceps-assisted removal may represent a useful option in selected cases in which the epithelial proliferation is accessible through the original incision, but further clinical experience and longer-term follow-up are required to assess its reproducibility, complications, and recurrence rate.

## Figures and Tables

**Table 1:** Published cases of epithelial ingrowth following keratorefractive lenticule extraction (KLEx/SMILE): a literature review.

Author, [Year]	N	Technique	Onset	Management/Surgical Timing	Postoperative Outcome	Last Follow-Up/Time to Resolution
Ahmet, S. [2023] [7]	1 (RE)	SMILE	1 week: epithelial inoculation following repeated inadvertent subepithelial dissector passage during loose-epithelium lenticule extraction	Interface irrigation with BSS via cannula (no scraping); performed immediately at diagnosis	1 day post-irrigation: complete opacity resolution UDVA 5/10, CDVA 10/10 (−1.00 −0.75 × 180°) Slit-lamp: smooth, hazy interface AS-OCT: no residual hyper-reflectivity	1 month: UDVA 10/10; slit-lamp and AS-OCT confirmed clear interface; no recurrence
Ding X. [2021] [9]	1	SMILE	20 days: asymptomatic strip-like opacity at routine follow-up (presumed intraoperative epithelial cell implantation)	Microforceps removal through the original SMILE incision; histopathological confirmation of corneal epithelial block; performed immediately at diagnosis	3 days post-removal: clear cornea UDVA 20/20	2 months: no recurrence; UDVA 20/20; transparent cornea
Kamiya K. [2020] [10]	2	Bilateral SMILE (incisional tear)	2 months (both cases): visually significant ingrowth after complicated lenticule extraction at an external center	Interface irrigation (BSS), blunt-spatula scraping, 27G vitreous forceps removal of epithelial tissue; no cap lifting 10-0 nylon sutures (×2) + therapeutic BCL ×1 week Treated at diagnosis	Case 1 at 1 month: MR +0.5 −1.0 × 180°, UCVA 1.2, BSCVA 1.5 Case 2 at 1 month: MR −1.75, UCVA 0.3, BSCVA 1.2	Case 1: no recurrence at 6 months Case 2: no recurrence at 3 months
Srivatsa S., Sood S. [2020] [11]	1	Bilateral ReLEx SMILE	1.5 years: incidental, non-progressive hyper-reflective interface lesion on AS-OCT; focal topographic flattening; no visual symptoms	Conservative management (observation); Nd:YAG laser and manual scraping discussed as options for progressive cases; no surgery performed	Asymptomatic; stable topographic and AS-OCT findings	6 months: stationary ingrowth; no progression; no intervention required
Vardhaman P. Kankariya [2021] [6]	3	SMILE	Case 1: visual loss onset 1 week post-op; presented at 3 months with UDVA 20/200, CDVA 20/80 Case 2: visual loss onset 2 months post-op; progressive worsening; surgery at 9 months Case 3: details not available	Cap-to-flap conversion via CIRCLE software (Zeiss VisuMax); complete mechanical debridement of stromal bed and flap undersurface; thorough interface irrigation	Data not available	Complete resolution; no recurrence (exact follow-up duration not specified)
Dhar S. [2026] [12]	1	SMILE	Progressive visual loss from 4 months post-SMILE; surgery performed at 11 months due to progressive worsening	Cap-to-flap conversion (CIRCLE software); stromal debridement + adjuvant MMC 0.02%; therapeutic BCL postoperatively	Day 1: UCVA 20/60, BCVA 20/20P (−1.25 DC × 125°); corneal regularization achieved Day 7: UCVA 20/40, BCVA 20/20P	5 months: complete recovery; no recurrence at 6-month follow-up
Zhang M. [2024] [13]	4	SMILE	Ingrowth associated with intraoperative OBL formation generating microchannels through Bowman’s layer (mechanistic characterization study)	AS-OCT and corneal tomography characterization; preventive intraoperative strategies proposed; detailed surgical management not reported	Multimodal characterization (AS-OCT, corneal tomography) of 4 cases; prevention-focused conclusions	No recurrence reported; follow-up duration not specified
Thulasi P. [2015] [5]	1	SMILE (complicated extraction)	8 months: visually significant recalcitrant ingrowth; two prior failed interventions	1st: scraping + debridement (shortly after diagnosis) → recurrence 2nd: scraping + flap-edge suturing → recurrence 3rd: scraping + hydrogel ocular sealant (ReSure)	Resolution of ingrowth and reduction in corneal haze after 3rd procedure; incidental diagnosis of uncontrolled diabetes (considered risk factor)	No recurrence after 3rd procedure + sealant; follow-up duration not specified

BCL = bandage contact lens; BCVA = best-corrected visual acuity; BSS = balanced salt solution; CDVA = corrected distance visual acuity; MMC = mitomycin C; MR = manifest refraction; OBL = opaque bubble layer; RE = right eye; SMILE = small incision lenticule extraction; UCVA = uncorrected visual acuity; UDVA = uncorrected distance visual acuity. N/A = not applicable.

## Data Availability

The original contributions presented in this study are included in the article/Supplementary Materials. Further inquiries can be directed to the corresponding author.

## Supplementary Materials

The following supporting information can be downloaded at: https://www.mdpi.com/article/10.3390/jcm15176928/s1, Video S1: Intraoperative video showing surgical removal of massive epithelial ingrowth following SMILE in the left eye. The epithelial tissue is delicately dissected and extracted using crocodile forceps under high magnification. Immediate interface clarity is achieved.

## Abbreviations

The following abbreviations are used in this manuscript:

AS-OCT	Anterior segment optical coherence tomography
KLEX	Keratorefractive lenticule extraction
SMILE	Small incision lenticule extraction
UCVA	Uncorrected visual acuity
